# Attachment of the levator ani muscle extends to the superior ramus of the pubic bone through electrophysiological and anatomical examinations

**DOI:** 10.1038/s41598-021-89041-6

**Published:** 2021-05-04

**Authors:** Hung-Yen Chin, Chih-Wei Peng, Ming-Ping Wu, Chih-Hwa Chen, Yu-Ting Feng, Tsorng-Harn Fong

**Affiliations:** 1 Department of Obstetrics and Gynecology, Taipei Medical University Hospital, School of Medicine, College of Medicine, Taipei Medical University, Taipei, Taiwan; 2 Department of Physical Medicine and Rehabilitation, School of Biomedical Engineering, College of Biomedical Engineering, Taipei Medical University, Taipei, Taiwan; 3 Division of Urogynecology, Department of Obstetrics and Gynecology, Chi Mei Medical Center, Tainan, Taiwan; 4 Department of Orthopedics, Taipei Medical University Hospital, Shuang Ho Hospital, School of Medicine, College of Medicine, Taipei Medical University, Taipei, Taiwan; 5 School of Biomedical Engineering, College of Biomedical Engineering, Taipei Medical University, Taipei, Taiwan; 6 Department of Early Childhood Care, Kun Shan University, Tainan, Taiwan; 7 Department of Anatomy and Cell Biology, School of Medicine, College of Medicine, Taipei Medical University, No. 250, Wuxing St, Xinyi District, Taipei, 11031 Taiwan

**Keywords:** Anatomy, Health care

## Abstract

Myofascial pelvic pain (MFPP) of pelvic floor muscles is a common cause of chronic pelvic pain (CPP). The pathological mechanisms and treatments of MFPP are complex and still unclear until now. The levator ani muscle (LAM) is the major pelvic floor muscle. The purpose of this study was to examine the fascia and attachment of LAM through the electromyogram (EMG) and cadaver dissection. Electrophysiological stimulation of the obturator fascia above the arcus tendinous levator ani (ATLA) could trigger contraction and electrophysiological changes in LAM insertion. The LAM of embalmed adult cadavers was examined especially in the area above the ATLA. Some skeletal muscle fibers were found above the ATLA within the obturator fascia and were confirmed by Masson’s trichrome section staining. Our electromyography (EMG) and anatomical data implied that the attachment of LAM aponeurosis extended beyond ATLA to the inferior border of the superior ramus of the pubic bone. The new discovered attachment of LAM could provide a reference position for clinical diagnosis and treatment of MFPP or CPP.

## Introduction

Chronic pelvic pain (CPP), defined as persistent, noncyclic pain in structures related to the pelvis and lasting more than 6 months, is a complex condition that can have multiple etiologies ranging from regional pathophysiologic pain to psychosocial aspects^1,2^. The causes of CPP may be associated with visceral or musculoskeletal dysfunctions, or may be associated with emotional, behavioral, sexual, and social consequences^3,4^. Among these etiologies, the musculoskeletal dysfunctions are of the most common cause for patients with CPP^5^.

Myofascial pelvic pain (MFPP), which is a frequent cause of CPP, refers to pain in the pelvic floor muscles, connective tissue, and surrounding fascia^6^. MFPP is estimated to affect 14–23% of women with CPP^7^. The symptoms of MFPP are similar to that of nonpelvic myofascial pain, such as back, neck, or shoulder pain^7^. Intralevator injection of botulinum toxin type A could improve pain in women with refractory MFPP^8^. Our previous study revealed that for MFPP management, diclofenac potassium provides better pain relief than other nonsteroidal anti-inflammatory drugs (NSAIDs)^9^. Treatments for MFPP include physical therapy, oral medications, cognitive-behavioral therapy, and botulinum toxin injections^10^. Moreover, myofascial physical therapy could reduce pain in CPP syndrome^11^. More studies are needed to provide diagnose of MFPP and determine the best course of treatment.

The levator ani muscle (LAM) constitutes the main part of the pelvic floor. It forms from the confluence of three muscles: puborectalis, pubococcygeus, and iliococcygeus muscles. Puborectalis originates from the inferior part of the pubic symphysis. Pubococcygeus originates from the posterior region of the inferior rami of the pubis and anterior part of the obturator fascia^12,13^. The arcus tendinous of the levator ani (ATLA), also known as the second white line, is the origin of iliococcygeus muscle^14^.

CPP and MFPP are often associated with disorders of muscle, bone or fascia in pelvic region^15,16^. LAM is the most important muscle that constitutes the pelvic floor. Due to improvements in the management of MFPP, it is necessary to examine the structure and boundary of LAM in more detail. Therefore, the objective of this study was to examine the attachment of the LAM by electromyography (EMG) and cadaver dissection. These results could provide a new perspective for clinical diagnosis and treatment of pelvic pain.

## Results

### Stimulation of the obturator fascia induced contraction and electrophysiological changes in the LAM

**Figure 1:**
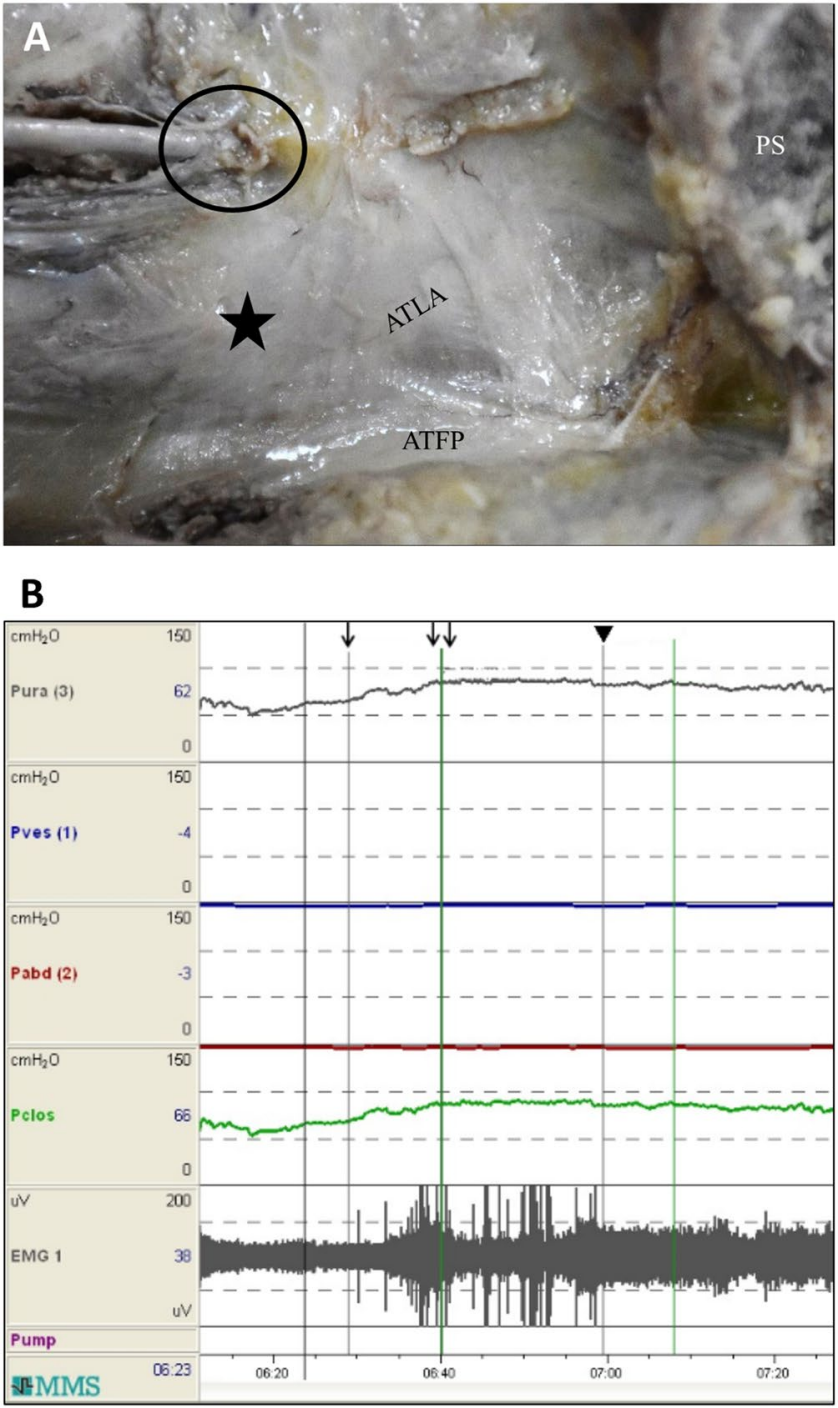
Electrode locations and electrophysiological changes in the levator ani muscle. (**A**) St. Mark’s disposable pudendal electrode was placed near the asterisk (*), which is near the posterior area of the obturator foramen above the arcus tendinous levator ani (ATLA). Detection patches were pasted at the bilateral perineal body near the anus. (**B**) Continuous cystometric and electromyogram (EMG) measurements obtained during the muscle stimulation test. When a low-intensity electrical current (15 mA) was delivered to the obturator fascia (at the single arrow site), the EMG activity gradually increased tonic amplitude, and some spontaneous spikes appeared during this period; simultaneously, an increase was observed in urethral pressure (Pura) and urethral closure pressure (Pclos) channels. Conversely, no drastic change was observed in the channels of vesical pressure (Pves) and abdominal pressure (Pabd). Subsequently, the electrical current intensity was increased to 30 mA (at the double arrow site), the tonic EMG and the rising pressures of Pura and Pclos immediately reached a plateau. The period of pressure plateau gradually decreased after the stimulation current was stopped (at the arrowhead site). The obturator canal is labeled by a circle. *ATFP* arcus tendineus fasciae pelvis, *PS* pubic symphysis.

For the electrical stimulation test, to identify whether the obturator fascia comprised muscle fiber components, the tip of the disposable pudendal electrode was used to target the obturator fascia through the vaginal outlet. The muscle stimulation test was conducted during continuous cystometric and EMG measurements. The stimulation points located in the cranially inferior area of the obturator foramen were reached by using the transvaginal approach (Fig. 1A), and the patches were placed at the bilateral perineal body near the anus. The typical pattern of cystometric and EMG activity with concomitant obturator fascia stimulation was shown in Fig. 1B. Initially, a low-intensity electrical current (15 mA) was continuously delivered to the fascia tissue, and the tonic EMG activity (the fifth channel from top) was gradually increased. This was mixed with some intermittent spontaneous spikes initially (single arrow indicates the start of stimulation with 15 mA); at this time, urethral pressure (Pura, the first channel from top) and urethral closure pressure (Pclos, the fourth channel from top) channels increased. Conversely, no drastic change was observed in vesical pressure (Pves, the second channel from the top) and abdominal pressure (Pabd, the third channel from the top) channels. Consequently, the stimulation amplitude was elevated to 30 mA (sites indicated by double arrows), and the spikes of EMG and the rising pressures of Pura and Pclos immediately reached a plateau. Consequently, the number of spontaneous EMG spikes further increased (Fig. 1B). These results indirectly demonstrated that the obturator fascia may include some muscle fibers instead of consisting of connective tissue only.

### Margins and thickness of the obturator fascia

**Figure 2:**
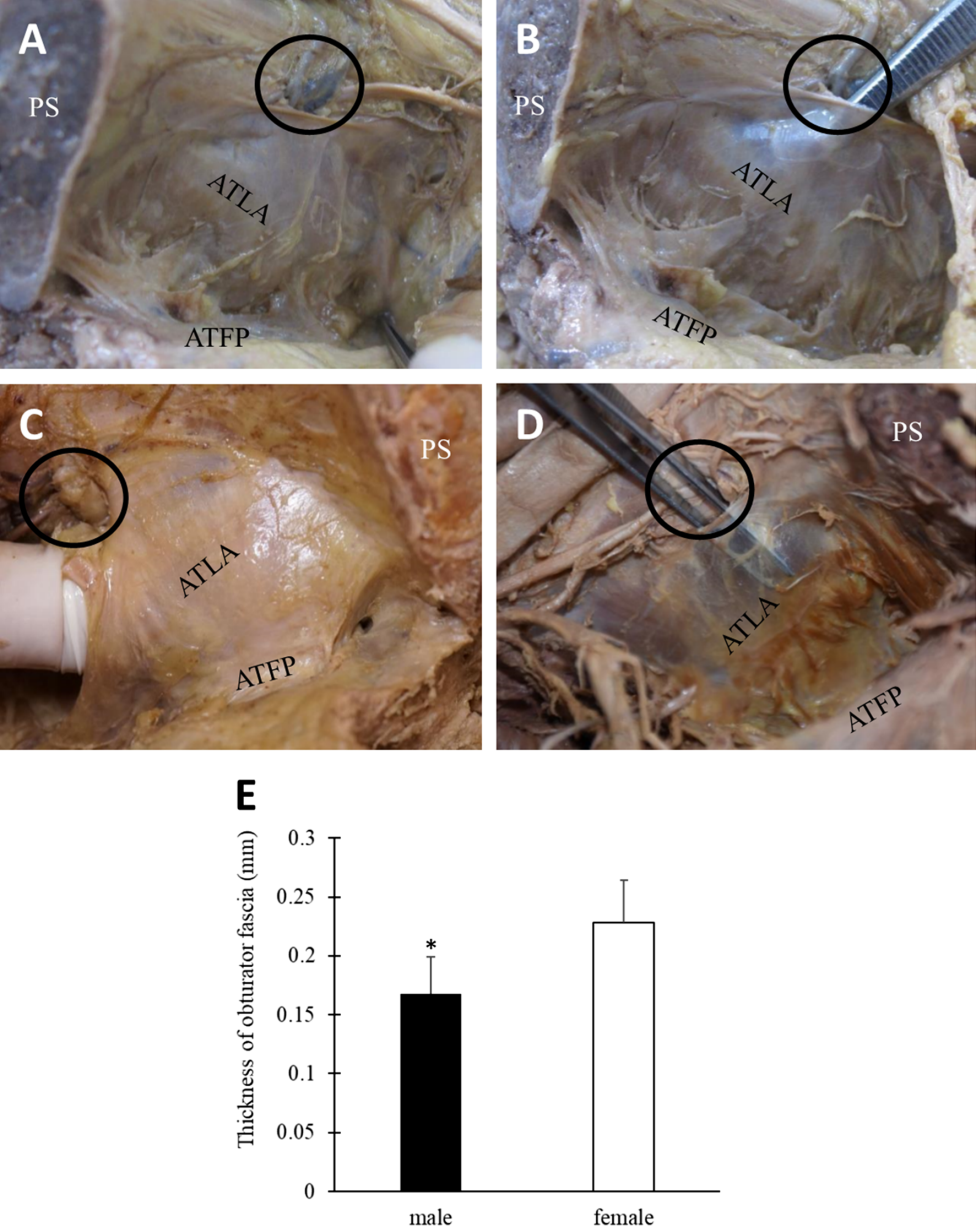
Photographs of the inner side of a hemipelvis with the intact obturator fascia. (**A**) The arcus tendinous levator ani (ATLA) is located within the obturator fascia and attached from the ischial spine to the inferior border of the superior ramus of the pubic bone. The arcus tendineus fasciae pelvis (ATFP) is located between the urinary bladder and obturator fascia and is attached from the ischial spine to the inferior border of the pubic symphysis (PS) in the right pelvis. (**B**) The obturator fascia can be separated from the obturator internus muscle through the obturator canal in the right pelvis. (**C**) The margins of the obturator fascia can also be identified through finger insertion into the space between the obturator fascia and obturator internus muscle in the left pelvis. (**D**) The obturator fascia in the left pelvis of male. (**E**) The thickness of obturator fascia of male is significantly thinner than that of female. The obturator canal is labeled by a circle. *p < 0.01.

In order to check the relationship of structure between obturator fascia and LAM in detail, we performed gross anatomy. Dissection of the pelvic cavity in the midline revealed an endopelvic fascia cover on the ventral surface of the urethra and bladder and the pelvic wall. The obturator fascia is located on the lateral side of the pelvic cavity and covers the obturator internus muscle (OIM). At the top of the obturator fascia is the obturator canal through which the obturator nerve and vessels pass. In addition, the ATLA is attached to the ischial spine and pubic bone within the obturator fascia (Fig. 2A). The obturator fascia could be separated from the OIM through the obturator canal (Fig. 2B). Visual examination of obturator fascia margins revealed that this fascia was superiorly and firmly attached to the inferior border of the superior ramus of pubic bone, posteriorly attached to the anterior border of the greater sciatic notch, inferiorly attached to the ischial spine and arcus tendineus fasciae pelvis (ATFP), and anteriorly fused with the fascia of the puborectalis muscle. We could open the obturator fascia through the obturator canal, and by placing a finger into the space between the obturator fascia and OIM, we could clearly identify the margins of the obturator fascia (Fig. 2C). Examination of the margins of the obturator fascia revealed that obturator fascia fused with the fascia of puborectalis muscle (anterior part of LAM).

In addition, we compared the thickness of obturator fascia between male and female cadavers (Fig. 2D). The quantitative results demonstrated that the average thickness of obturator fascia in male (0.167 ± 0.031 mm) was thinner than in female (0.228 ± 0.036 mm) (Fig. 2E, p < 0.01, t-test).

### The obturator fascia contains skeletal muscle fibers of LAM above the ATLA

**Figure 3:**
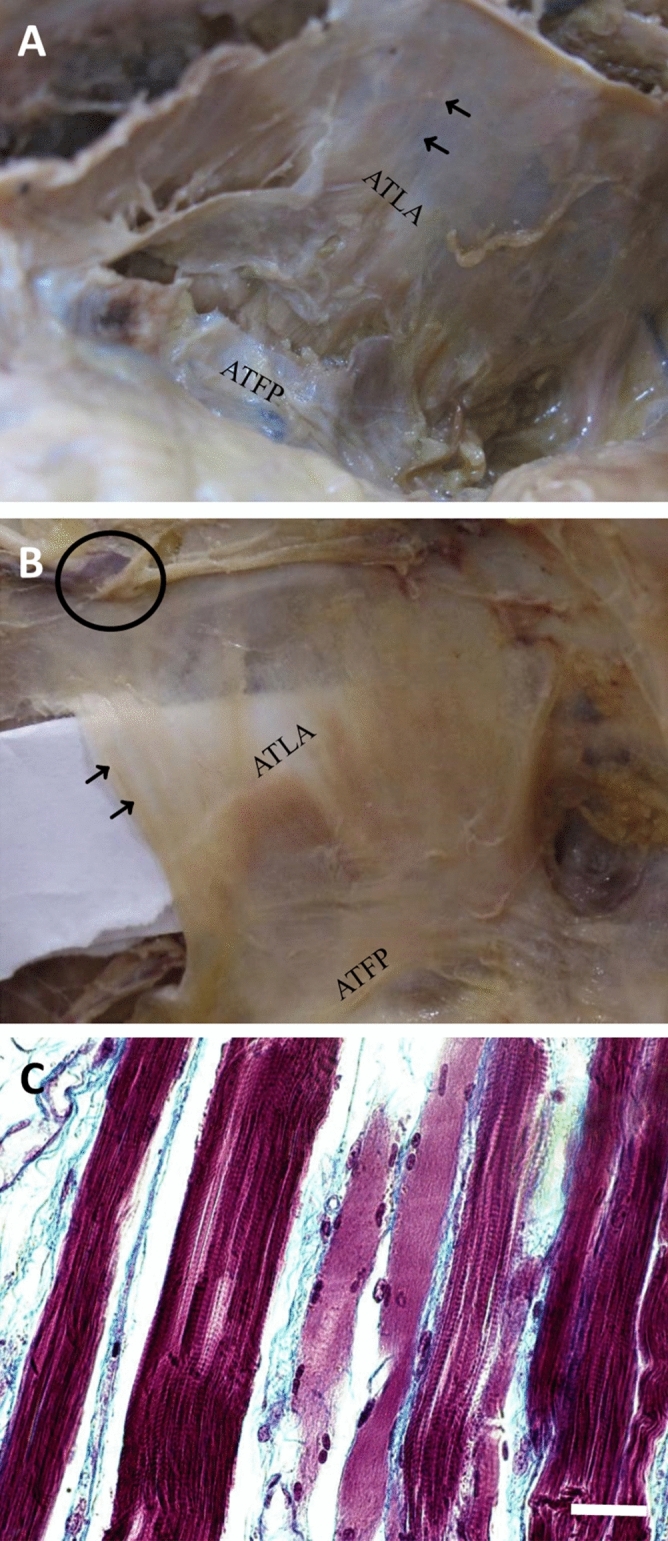
Photographs of the inner side of a hemipelvis displaying muscle fibers within the obturator fascia. (**A**) Some muscle fibers (arrows) can be observed within the obturator fascia above the arcus tendinous levator ani (ATLA) in the right pelvis. (**B**) These muscle fibers (arrows) could be clearly observed by placing a white piece of paper between the obturator fascia and obturator internus muscle in the left pelvis. (**C**) Histological section revealing muscle fibers within the obturator fascia are skeletal muscle type. These striated muscle fibers are stained in red, and the abundant collagen fibers of the connective tissue between muscle fibers are stained in blue/green through Masson’s trichrome staining. The obturator canal is labeled by a circle. *ATFP* arcus tendinus fasciae pelvis. Bar, 20 μm.

When we checked obturator fascia by the incision and reflection of the obturator fascia through the obturator canal, we found some muscle fibers above the ATLA in the obturator fascia (Fig. 3A). The direction of fascia muscle fibers was distinct from that of the OIM, suggesting that the thin muscle fibers did not belong to the OIM.

We traced the direction of the muscular fibers in the obturator fascia and found that these were extensions of the LAM. This suggested that the muscle fibers of the LAM were distributed not only below but also above the ATLA within the obturator fascia. We could observe the muscle fibers in the fascia clearly after placing a white piece of paper between the obturator fascia and OIM (Fig. 3B).

The fascia containing the thin layer of muscle fibers was isolated, and histological examination was performed. Masson’s trichrome staining confirmed that the muscle fibers within the obturator fascia above the ATLA were striated skeletal muscles (Fig. 3C). These results show that the obturator fascia contains some skeletal muscle fibers of the LAM above the ATLA.

### The fascia of the LAM was fused with the obturator fascia above the ATLA

**Figure 4:**
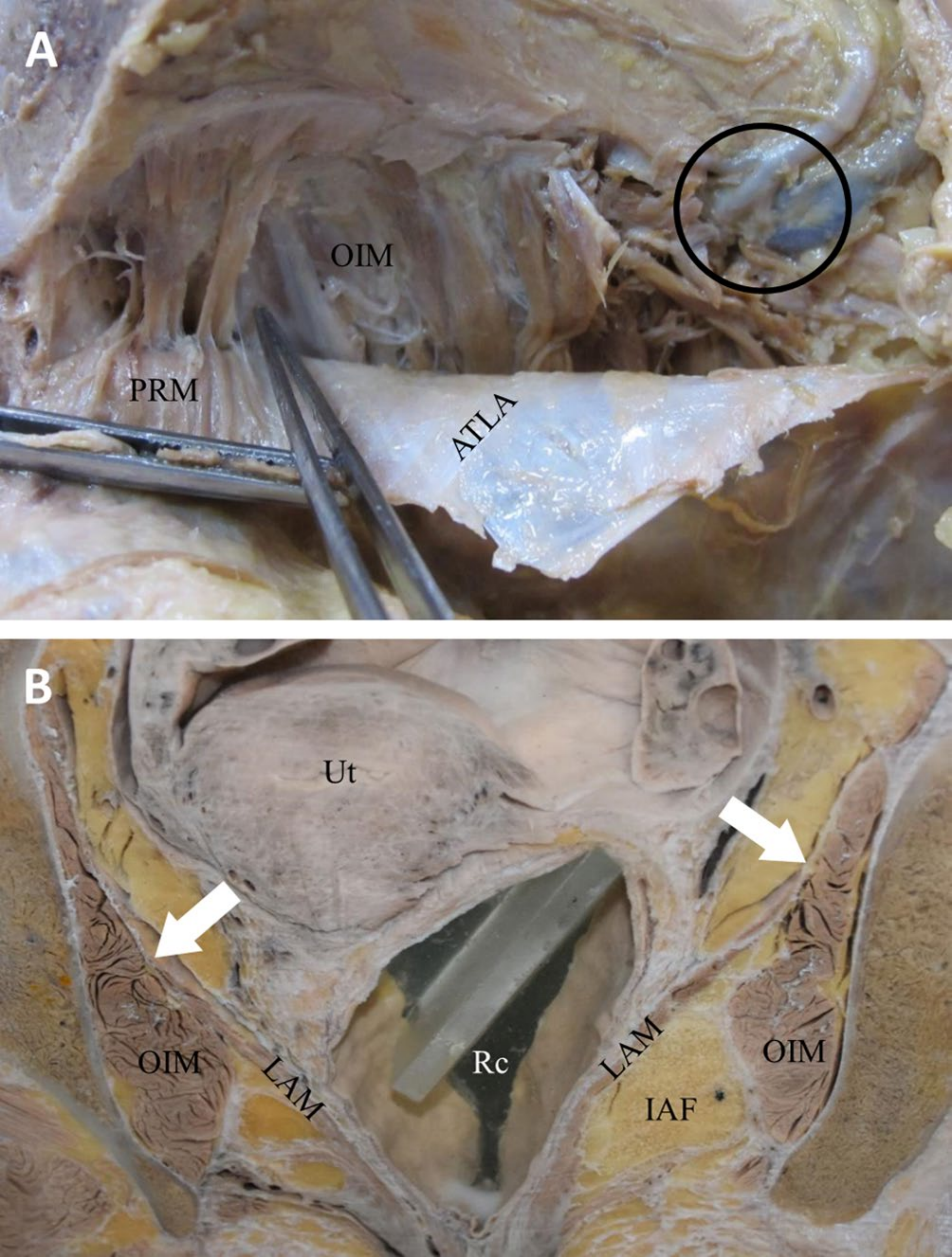
Photographs presenting the levator ani muscle (LAM) fascia fused with the obturator fascia above the arcus tendinous levator ani (ATLA). (**A**) Photographs of the right side of the hemipelvis with a reflected obturator fascia and an exposed obturator internus muscle (OIM). Detaching the obturator fascia would detach puborectalis muscle (PRM) of the LAM and destroy the muscle fibers of the OIM only above the ATLA. (**B**) Whole-body frontal section displaying the fascia of the LAM fused with the obturator fascia above the ATLA. Arrows indicate the fusion site of the LAM fascia with the obturator fascia. The obturator canal is labeled by a circle. *IAF* ischioanal fossa, *Rc* rectum, *Ut* uterus.

Reflecting the obturator fascia from the obturator canal would destroy the muscle fibers of the OIM above the ATLA but not the muscle fibers below the ATLA (Fig. 4A). Thus, the obturator fascia was firmly attached to the OIM above the ATLA. In addition, reflecting the anterior part of the obturator fascia could detach the origin of puborectalis muscle, which is the anterior part of the LAM (Fig. 4A). Thus, the fascia of the LAM may be fused with the obturator fascia above the ATLA.

Examination of the pelvic region of whole-body frontal sections revealed that the ischioanal fossa is located between the LAM and OIM, and the fascia of the LAM was fused to the obturator fascia above the ATLA (Fig. 4B). These results suggested that the ATLA was not the site of the LAM origin but was the fusion line of the LAM fascia with the OIM fascia.

## Discussion

According to EMG and anatomy data, the LAM aponeurosis may extend beyond ATLA to the inferior border of the superior ramus of the pubic bone. Gross anatomical and histological examinations showed a thin skeletal muscle layer within the obturator fascia above the ATLA. Furthermore, EMG results suggested that this skeletal muscle tissue within the obturator fascia has neuromuscular relations and acts as a functional unit of the LAM complex. The attachment of LAM fascia could extend to the pubic bone and might provide another approach for the clinical diagnosis and treatment of CPP and MFPP.

The EMG muscle stimulation experiment showed that the electrical stimulation of the obturator fascia could activate tonic and spike EMG activity of the electrode located on the perineal body, a secondary insertion of the LAM. This offered evidence that the obturator fascia might consist of some striated muscle fiber components of the LAM. Although surface EMG technology has some limitations compared with needle electrodes, it is still suitable for studying the physiological characteristics of the LAM because of its simplicity and noninvasive methodology for the effective activation of LAM^17^. Therefore, we used surface pudendal electrodes for muscle stimulation testing. We delivered the stimulation current starting from the lowest intensity and gradually increased the intensity until muscle contraction was detected. The distribution area of stimulation current on the vaginal muscle mainly depended on the distance between the electrode and target muscle tissue. At the lowest stimulation intensity, the electrical current only evoke the target muscle beneath the electrode–tissue area due to its close proximity; however, we did not trigger obturator internus muscle contraction, and EMG activity can be detected on the perineal body electrodes, implying LAM attachment from the obturator fascia to perineal body.

The gross anatomical and histological examination revealed that the obturator fascia above ATLA contained some skeletal muscle fibers of LAM. When we separated obturator fascia from OIM through obturator canal, we found that obturator fascia was connected with LAM and form an integral structure. These anatomical results provided evidence for proving that the scanty muscle fibers within obturator fascia were continuous with muscle fibers of LAM. On the other hand, LAM is a flat muscle of pelvic floor, similar to the flat muscles of abdominal wall. It is well known that the aponeurosis of three abdominal muscles fuse together to form the rectus sheath and then end in linea alba^18^. There are some muscle fibers of LAM in obturator fascia above ATLA, indicating that the aponeurosis of LAM might fuse with obturator fascia and the fusion line was the ATLA.

The origin of puborectalis is the inferior part of the pubic symphysis, and the origins of pubococcygeus and iliococcygeus are the ATLA^14^. Our findings suggested that the origin of pubococcygeus and iliococcygeus might extend from ATLA to the inferior border of superior ramus of pubic bone. The origin of three parts of LAM might be continuous from pubic symphysis to inferior border of superior ramus of pubic bone. We speculated that three parts of LAM that are attached to the pubic bone should provide stronger support for pelvic organs than just being attached to the ATLA (a tendinous arch).

All the cadavers we dissected showed that the aponeurosis of LAM fused with obturator fascia above the ATLA. In addition, we compared the features of obturator fascia between males and females. We found that the thickness of fused obturator fascia was thicker in females than in males, and the skeletal muscle fibers within obturator fascia were more obvious in females than in males. 3D topography reconstructed from serial anatomical sections demonstrated that the volume of the LAM in females was approximately two-fold larger than in males^19,20^. These above-mentioned features probably indicated a higher workload of LAM in females than in males.

MFPP is an under-diagnosed syndrome and an untreated component of CPP. A levator ani muscle trigger point injections with a mixture of bupivacaine, lidocaine, and triamcinolone can effectively reduce pain in women with CPP and specific palpable levator ani trigger points^21^. In addition, an enthesopathy, such as fasciitis and tendinitis, is an enthesis-related disorder, which involves the attachment of a tendon or ligament to a bone^22,23^. There is currently no literature mentioning injection the inferior border of superior ramus of pubic bone for treatment of MFPP, CPP, or enthesopathy of pubic bone. However, our study suggested that the bone attachment of the LAM could provide a reference position for injection or treatment.

The strength of this study is that we could examine the attachment of LAM with more detail using cadaver dissection. However, the limitations of our study are as follows: first, small number of cadavers, second, lack of sufficient healthy participations to provide clinical information of EMG. We offered a new anatomical discovery about the boundary and attachment of LAM in this study.

In conclusion, the EMG as well as morphological and histological examinations showed that the LAM aponeurosis extended beyond ATLA to the inferior border of the superior ramus of the pubic bone. The obturator fascia fused with the LAM aponeurosis above the ATLA. CPP is a difficult condition to evaluate and treat. We suggested that the clinical diagnosis and treatment of pelvic pain may consider the boundary of LAM. It is worth conducting more clinical researches to re-examine the levator ani syndrome in future studies.

## Materials and methods

### Study participants

A total of 10 women (4 of whom were multiparous) aged 23–70 years with over activity bladder syndrome were enrolled in the study. They all met the indication of urodynamic examination, and all voluntarily participated in the electrophysiological muscle stimulation test. None of the participants had a history of pelvic surgery or neuropathic disease to eliminate possible confounding factors in the studies of nerve conduction. We did not enroll the CPP patients for muscle stimulation test because they might have varying degrees of levator ani muscle pathology. In addition, ten embalmed adult cadavers (four female and six male) were recruited for gross dissection and histologic examination.

The protocols of human experiment were approved by the Taipei Medical University Joint Institutional Review Board (No. N201712008). In addition, the gross anatomical study was approved by the Taipei Medical University Joint Institutional Review Board (No: 201707048), Taipei, Taiwan. We obtained informed consent from all patients by themselves before they were enrolled. All experiments were performed in accordance with relevant named guidelines and regulations.

### Muscle stimulation test

St. Mark’s disposable pudendal electrodes (Alpine Biomed, Skovlunde, Denmark) were fitted over the index finger of a disposable glove, with the stimulating anode and cathode positioned at the fingertip. Points located in the cranially inferior area of the obturator foramen above the ATLA were touched using the transvaginal approach, and biphasic stimulation current pulses were produced by a stimulator (EM-9000, Everyway Medical Instruments Co., New Taipei City, Taiwan) using a transcutaneous electrical nerve stimulation model. The stimulation frequency was fixed at 80 Hz (pulse width: 180 μs). The pulse current was adjusted from 0 to 30 mA depending on the positive response observed on the electromyography monitor.

Before the formal muscle stimulation experiment, each subject was presented with a series of electrical stimulation pre-tests surrounding the target area of levator ani muscle on the fascia to screen out the optimal hotspot site. Generally, when the pulse current was adjusted at low intensities (from 0 to ~ 15 mA), there was no EMG response. Accordingly, the distribution area of stimulation current on the vaginal muscle mainly depends on the distance between the electrode and target muscle tissue. At the lowest stimulation intensity, the electrical current would only evoke the target muscle beneath the electrode–tissue area due to its close proximity; however, EMG activity can be detected on the perineal body electrodes.

The EMG and intravesical pressure signals of stimulation trials were simultaneously collected by our urodynamic system (UD-2000, Medical Measurement Systems B.V., Netherlands). The patches were pasted at the bilateral perineal body near the anus.

### Gross anatomical dissection

Gross dissection was performed on ten embalmed adult cadavers, four of which were female specimens (aged 64, 68, 82, and 85 years) and six were male specimens (aged 39, 73, 74, 82, 85, and 86 years). No history of pelvic surgery was recorded for these ten cadavers. Within 6 h of death, the cadavers were perfused through the femoral artery under hydrostatic pressure with a fixative solution containing 50% ethanol, 10% formalin, 7% glycerin, and 7% phenol in water. After perfusion, the cadavers were stored in a 10% formalin solution for 12–18 months.

During the dissection, abdominis muscles were removed first to open the pelvic cavity. This was followed by sawing through the pubic symphysis, sacrum, and coccyx in the mid-sagittal plane. The peritoneum and fat tissue between the urinary bladder and pelvic wall were removed until the lateral pelvic wall was clearly visible.

The thickness of obturator fascia was measured by using digimatic micrometer (Mitutoyo Corp., Kanagawa, Japan). The obturator fascia was detached from the superior ramus of pubic bone, and then we measured the middle position between obturator canal and ischial spine.

### Histological sections

We performed light microscopy to examine the muscle tissue in the obturator fascia. The samples were harvested and postfixed overnight with 4% paraformaldehyde in phosphate buffer (pH 7.4). Subsequently, the samples were dehydrated in an ethanol series, which was followed by xylene infiltration and paraffin embedding by using standard procedures. Sections of 5-μm thickness were cut and stained with Masson’s trichrome stain, and then examined under a microscope (Nikon Co., Tokyo, Japan).

### Examination of whole-pelvis sections

Serial whole-pelvis sections of adult cadavers (purchased from Foremost Scientific Co., Taipei, Taiwan) were studied. Cadavers were perfused through the femoral artery under hydrostatic pressure with a fixative solution containing 50% ethanol, 10% formalin, 7% glycerin, and 7% phenol in water. After perfusion, cadavers were frozen in dry ice and then cut with a band saw at 2-cm intervals in frontal planes. Each section was stored in a plastic box filled with 10% formalin.

### Statistical analysis

Values are expressed as the mean ± SEM. Student's *t*-test was used to verify the significance of the differences between male and female, and p < 0.01 was considered significant.
